# Stem cells derived from human first-trimester umbilical cord have the potential to differentiate into oocyte-like cells *in vitro*

**DOI:** 10.3892/ijmm.2015.2132

**Published:** 2015-03-11

**Authors:** XIANG HU, HUA LU, SHENG CAO, YAN-LI DENG, QI-JIA LI, QIAN WAN, SHANG-MIAN YIE

**Affiliations:** Department of Gynecology and Obstetrics, The Second Medical College/Teaching Hospital, Chengdu University of Traditional Chinese Medicine, Chengdu, Sichuan 610041, P.R. China

**Keywords:** stem cells derived from human first-trimester umbilical cord, primordial germ cells, oocyte-like cells

## Abstract

Compared to stem cells derived from human term umbilical cord, stem cells derived from human first-trimester umbilical cord (hFTUC) exhibit a significantly greater proliferative potential, and more efficiency in terms of their *in vitro* differentiation. In the present study, we investigated whether hFTUC-derived stem cells are able to differentiate into germ cells. The hFTUC-derived stem cells were first isolated, expanded and then cultured in differentiation medium containing human follicular fluid, follicle-stimulating hormone (FSH)/luteinizing hormone (LH) and estradiol for 24 days. During the period of induction, a subpopulation of the cultured cells appeared that had a morphological resemblance to primordial germ cells (PGCs) and cumulus-oocyte complex (COC)-like cells, and oocyte-like cells (OLCs). The PGC-like cells expressed specific markers indicative of germ cell formation such as octamer-binding transcription factor 4 (OCT4), stage-specific embryonic antigen 1 (SSEA1), B lymphocyte-induced maturation protein-1 (BLIMP1), PR domain containing 14 (PRDM14), transcription factor AP-2 gamma (TFAP2C), VASA, STELLA, deleted in azoospermia-like (DAZL) and interferon-induced transmembrane protein 3 (IFITM3). The OLCs, which contained a single germinal vesicle, expressed oocyte-specific markers, such as synaptonemal complex protein 3 (SCP3), growth/differentiation factor-9 (GDF9), GDF9B and zona pellucida (ZP)1, ZP2 and ZP3. The COC-like cells secreted estradiol, vascular endothelial growth factor and leukemia inhibitory factor. Thus, our findings suggest that hFTUC-derived stem cells have an intrinsic ability to differentiate into OLCs, which may provide an *in vitro* model for the identification of factors involved in germ cell formation and differentiation.

## Introduction

Germ cells are the biological route for the transmission of genes from one generation to the next. With their unique characteristics, germ cells constitute a cell population which is very different to somatic cells, and display a haploid chromosomal number following a delicate process of meiosis (1). However, important questions regarding how germ cells are specified remain unanswered in human and mammalian developmental biology.

The generation of germ cells from somatic cells *in vitro* may provide a valuable model for identifying factors involved in germ cell formation and differentiation. Accordingly, numerous attempts have been made over the past decade to determine whether murine or human embryonic stem (ES) cells are able to differentiate into primordial germ cells (PGCs) or oocyte-like cells (OLCs) *in vitro* (2–7). Moreover, it has been reported that germ cell-like cells can be derived *in vitro* from multipotent stem cells derived from newborn mice or porcine fetal skins (8–10), mesenchymal stem cells (MSCs) derived from mouse bone marrow (BM) (11), or human adult ovaries (12).

Additionally, certain studies have reported that human or murine ES cells can spontaneously differentiate into OLCs in adherent cultures or through embryoid body formations (5–7). Other studies have reported that human or mouse ES cells or multipotent stem cells other than ES cells can form germ-like cells and mature gametes by using various differentiation strategies, such as the addition of exogenous factors (6,13) or follicular fluid (8) to the culture medium, or by co-culture with ovarian granulose cells (3).

Previously, we isolated and characterized human first-trimester umbilical cord (hFTUC)-derived stem cells and found that the cells exhibited characteristics of pluripotent stem cells, including the expression of pluripotent stem cell markers, such as octamer-binding transcription factor 4 (OCT4), Nanog, (sex determining region Y)-box 2 (SRY, also known as SOX2), stage-specific embryonic antigen (SSEA)3, SSEA4, Tra-1-60 and Tra-1-81, as well as formations of embryoid bodies (14). Furthermore, we found that hFTUC-derived stem cells exhibited a significantly greater proliferative potential, and were more efficient in their *in vitro* differentiation toward selective mesenchymal cell types, including chondrogenic and adipogenic lineages, as well as neuronal- and hepatocyte-like lineages (15). Thus, we hypothesized that hFTUC-derived stem cells may have an intrinsic ability to form germ cells and differentiate into OLCs *in vitro*. In the present study, we examined this hypothesis by first isolating and expanding hFTUC-derived stems cells, and then inducing the differentiation of cells using differentiation medium. Subsequently, we analyzed the cells for their morphological appearance, the expression of markers indicative of germ cell formation and oocyte development, as well as estradiol production.

## Materials and methods

### Isolation and culture of hFTUC-derived stem cells

The isolation and expansion of the hFTUC-derived stem cells were based on a method employed in a recently published study of ours (15). Briefly, first-trimester umbilical cords were collected following therapeutic pregnancy interruptions, which were carried out with the written informed consent of the patients and approval from the Second Medical College/Teaching Hospital Institutional Review Board, Chengdu University of Traditional Chinese Medicine, Chengdu, China. To isolate the stems cells, the cords were rinsed several times with sterile saline. In order to completely avoid of any contamination from maternal or fetal sources, the cells that were not attached to/or within the umbilical cord tissue at the beginning of the process were discarded during each rinsing. Subsequently, the cords were rinsed several times with sterile saline and cut into sections followed by an immersion in 1% collagenase type I (Sigma-Aldrich, St. Louis, MO, USA) solution for 1 h at 37°C. The denuded tissues were discarded following the removal of the cells during digestion. The cells were then pelleted by a low-speed centrifugation (250 × g for 5 min) and suspended in fresh medium. The cells were then plated into a 25 cm culture flask with an expansion medium (Minimum Essential Medium Eagle, Alpha Modification; α-minimal essential medium (α-MEM) (Sigma-Aldrich), which was supplemented with 10% fetal bovine serum (FBS), 2 mmol/l L-glutamine, 50 IU/ml penicillin and 50 mg/ml streptomycin (both from Gibco-BRL, Gaithersburg, MD, USA) in 5% CO_2_ at 37°C. The culture medium was replaced every 3 days. Subconfluent (70–80%) cells were detached with 0.05% trypsin-0.01% EDTA (Gibco-BRL) and plated at a density of 2.4×10^3^ cells/cm^2^.

### Karyotype analysis

Karyotype analysis of the hFTUC-derived stem cells was performed by using an AneuVysion Multicolor DNA Probe kit (Vysis CEP 18/X/Y-α-satellite/LSI 13/21) with DNA probes for chromosomes X and Y (Abbott Molecular Diagnostics, Des Plaines, IL, USA) in accordance with the manufacturer’s instructions.

Briefly, the cultured undifferentiated stem cells in culture-ware (BD Biosciences, San Jose, CA, USA) were fixed with 3:1 methanol:acetic acid and exposed to denaturing solution (70% formamide in 2X SSC pH 8.0) prior to hybridization. Subsequently, the X and Y probes were applied to the slides, which were immediately covered with coverslips and sealed. The slides were subsequently incubated in hybridization chamber for 6 h at 37°C. Following hybridization, the cover-slips were removed and the slides were then washed with a solution of 2X SSC/0.1% NP-40 followed by a solution of 2X SSC/0.1% NP-40. The slides were allowed to air-dry in the dark and DAPI II counterstain was performed before covering the slides with a glass coverslip. Finally, signal enumeration on the slides was visualized under a fluorescence microscope (Leica M205 FA) (Leica Microsystems, Wetzlar, Germany). In the present study, 6 hFTUC-derived stem cell lines were isolated, with 3 cell lines with the XX karyotype and 3 cell lines with the XY karyotype.

In our preliminary experiments, we did not find any significant difference in the efficiency of the induction of the stem cells into germ cell-like cells between the female and male umbilical cord cells. This result is similar to a recent report regarding the effect of gender on the differentiation of MSCs into germ cells (16). Thus, in order to avoid the effects of possible contamination by the cells of the mother, the XY karyotype cell line was used in the following experiments.

### Induction of differentiation

In order to induce differentiation into female germ cells, the hFTUC-derived cells at the 3rd passage were trypsinized, and 1–5×10^4^ cells/well were plated into culturewares or 6-well culture plates (BD Biosciences) and cultured in α-MEM containing 10% FBS, with or without 25% human follicular fluid (hFF), 150 mIU follicle-stimulating hormone (FSH), 150 mIU luteinizing hormone (LH) (Ferring Pharmaceuticals Inc., Parsippany, NJ, USA) and 300 pg/ml of estradiol (Sigma-Aldrich) at 37°C in an atmosphere of 5% CO_2_ air.

The culture medium was replaced every 3 days. The morphology of the cells was examined and images were captured using an inverted microscope (Leika DMI 3000B; Leica Microsystems) prior to the medium changes. At 7 days after differentiation, a subpopulation of cells showing a round shape became visible. The cells were collected and analyzed either by RT-qPCR, western blot analysis or immunofluorescence staining.

At 14 days after differentiation, cell aggregates resembling follicle-like structures were appeared. The cell aggregates were retained in this culture medium for a further 14 days by replacing the medium every 2–3 days. At 21–24 days after differentiation, OLCs varying in size from 50 to 120 *μ*m in diameter were observed. The OLCs were collected and analyzed either by RT-qPCR analysis, western blot analysis or immunofluorescence staining.

The culture medium was collected at each medium replacement and stored at −80°C until analysis to determine the production of estradiol, vascular endothelial growth factor (VEGF) and leukemia inhibitory factor (LIF).

### Immunofluorescence staining

The cells that were treated for 7 and 24 days with or without the differentiation medium were washed with phosphate-buffered saline (PBS) and fixed with 4% ice-cold paraformaldehyde for 10 min. Following 3 washes with PBS, the fixed cells were incubated for 1 h at room temperature with one of the primary antibodies in an antibody dilution solution (Dako Denmark A/S, Glostrup, Denmark). The source and dilution information of the primary antibodies are presented in Table I.

Following incubation with the primary antibodies and 3 PBS washes, the cells were incubated with FITC-conjugated goat anti-rabbit or rabbit anti-mouse antibodies (Sc2012; Santa Cruz Biotechnology, Inc., Dallas, TX, USA) diluted at 1:1,000 in PBS-3% (w/v) BSA for 1 h at room temperature. After washing another 3 times with PBS, the nuclei were stained with DAPI, and visualized under a fluorescence microscope (Leica M205 FA; Leica Microsystems).

Mouse oocytes, the maturation of which was promoted by treatment with pregnant mare serum gonadotrophin (PMSG), were used as the positive controls. Negative controls were prepared by the omission of the primary antibodies.

### RT-qPCR

Total cellular RNA was isolated using TRIzol^®^ reagent (Macherey-Nagel, Düren, Germany) according to the manufacturer’s instructions. Subsequently, 0.5 *μ*g of the total RNA was reverse transcribed into cDNA using a Superscript II Reverse Transcriptase kit (Fermentas Life Sciences, Schwerte, Germany) in accordance with the manufacturer’s instructions.

Quantitative (real-time) PCR (qPCR) was performed using the SYBR-Green mix kit on an ABI Prism 7900 sequence detector (both from Applied Biosystems, Foster City, CA, USA). A total of 2.0 *μ*l cDNA was added to 12.5 *μ*l SYBR-Green mix with 0.3 *μ*M each of the forward and reverse primers. Water was added to produce a final volume of 25 *μ*l. The reaction was carried out for 40 cycles of 95°C for 15 sec, 56–62°C for 30 sec (primer-dependent), 72°C for 30 sec, and a final cycle of 75°C for 30 sec. The primer sequences for OCT4, SSEA1, STELLA, VASA, B lymphocyte-induced maturation protein-1 (BLIMP1), PR domain containing 14 (PRDM14), transcription factor AP-2 gamma (TFAP2C), synaptonemal complex protein 3 (SCP3), growth/differentiation factor-9 (GDF9), ZP1, ZP2, ZP3 and β-actin are listed in Table II.

For each PCR product, the melting curve was determined using the comparative threshold cycle number (2^−ΔΔCt^) method, with the results being presented as the fold change in the expression of the genes in the cells induced to differentiate relative to the undifferentiated cells (controls), as previously described (17). All the experiments were performed in triplicate and were repeated at least 3 times on different occasions.

### Western blot analysis

To examine protein expression, the cells were collected using 200 *μ*l cell lysis buffer (50 mM Tris-HCl, pH 7.4, 150 mM NaCl, 1 mM PMSF, 1 mM EDTA, 1% Triton X-100 and 1% SDS). An equal protein concentration of cell lysates/lane (10 *μ*g/lane) was separated using 10% SDS-PAGE and electroblotted onto polyvinylidene fluoride (PVFD) membranes (Invitrogen, Carlsbad, CA, USA). The membranes were blocked in PBS containing 0.05% Tween-20 (PBS-T) and 5% skim milk for 2 h at room temperature, and then incubated with polyclonal rabbit anti-human OCT4, STELLA, VASA, GDF9, ZP1 (Table I) and anti-β-actin antibodies (Abcam, Cambridge, UK) overnight at 4°C. The membranes were washed 3 times (10 min/wash) with PBS-T, and incubated with a horseradish peroxidase-conjugated goat anti-rabbit antibody (1:2,000; Sigma-Aldrich) for 1 h at room temperature. The membranes were washed 3 times again and antigen-antibody complexes were visualized using tetramethylbenzidine (TMB) (Sigma-Aldrich).

### ELISA for estradiol, VEGF and LIF expression in the culture supernatant

The concentrations of estradiol, VEGF and LIF in the culture supernatant were determined using a specific estradiol ELISA kit (Catalog no. 1920) (Alpha Diagnostic International, San Antonio, TX, USA), a VEGF ELISA kit (Catalog no. DVE00) and a LIF ELISA kit (Catalog no. DLF00) (both from R&D Systems, Minneapolis, MN, USA). The assays were performed according to the respective manufacturers’ instructions. The intra- and inter-assay variations were 3.5–5.0 and 10.2–13.1%, respectively. ELISAs were performed in a blinded manner.

### Statistical analysis

Statistical analysis was performed using the SPSS software package (SPSS Inc., Chicago, IL, USA). Differences in the mRNA expression of the markers and the concentrations of estradiol, VEGF and LIF in the culture supernatant between the cells induced to differentiate and the undifferentiated cells were analyzed using the one-way analysis of variance. If significance was found in the analysis, the data underwent post-hoc comparisons. A value of P<0.05 was considered to indicate a statistically significant difference.

## Results

### Differentiated cells with morphological resemblance to PGCs and OLCs

**Figure 1:**
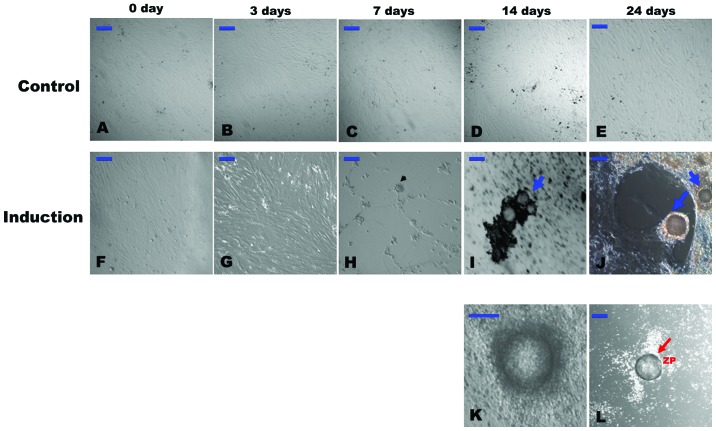
(A–E) Representative images of untreated cells (controls) cultured in α-minimal essential medium (α-MEM) containing 10% fetal bovine serum (FBS) from day 0–24, during which no significant morphological changes were observed. However, after 3 days of culture in the differentiation medium, some subpopulations of the cells appeared morphologically distinct from the starting culture (G). After 7 days of differentiation, primordial germ cell (PGC)-like cells, which ranged from approximately 15–20 *μ*m in diameter, appeared (H, arrowhead). After 14 days of differentiation, oocyte-like cells (OLCs) developed and were surrounded by smaller cells (I and J, blue arrows). Higher magnification images of OLCs are also presented to show that they are in pachytene (K). Occasionally, some of the OLCs were coated with a zona pellucida (ZP)-like structure (L, red arrow). Scale bar is set to 100 *μ*m.

As shown in our previous study, the hFTUC-derived stem cells can be propagated in long-term culture for at least 15 passages without the induction of differentiation (15). Similarly, the cells in the present study grew adherent, exhibited spindle-like shapes and resembled the morphological characteristics of undifferentiated fibroblast-like cells in α-MEM with 10% FBS during the 24 days of experiments (Fig. 1A–E).

However, after 3 days of differentiation, some subpopulations of the cells became morphologically distinct from the starting cultures (Fig. 1G). PGC-like cells, which ranged from approximately 15–20 *μ*m in diameter, appeared around at 7 days of differentiation (Fig. 1H). Aggregates were formed after 7 days of differentiation. These aggregates gradually became morphological structures similar to a primordial follicle after 14 days (Fig. 1I). At 14–24 days of differentiation, OLCs were developed much like those described in a previous study (Fig. 1J) (8). The OLCs became surrounded by smaller cells over time that resembled cumulus-oocyte complex (COC)-like cells (Fig. 1K). Occasionally, some of the OLCs were observed to be coated with a zona pellucida (ZP)-like structure (Fig. 1L), and some of the OLCs reached >100 *μ*m in diameter.

### Immunofluerence staining for the expression of markers related to germ cell formation and oocyte development

In order to verify whether the PGC-like cells and OLCs, that were identified based on morphological changes, expressed specific gene markers related to germ cell formation and oocyte development, we first performed immunofluerence staining of the cells at 7 and 24 days of induction, respectively.

**Figure 2:**
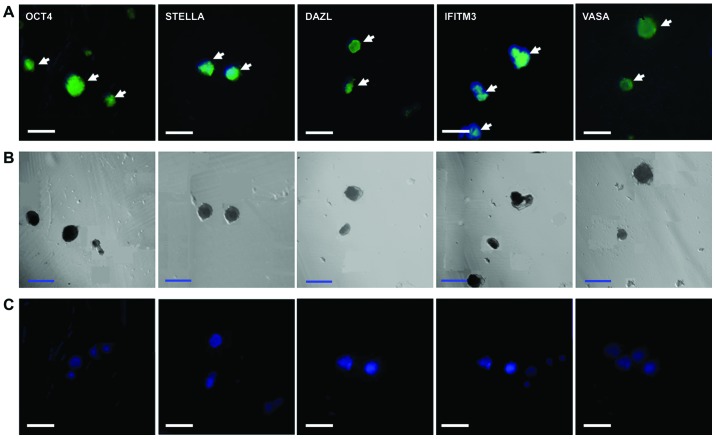
Immunofluorescence staining of germ cell markers in primordial germ cell (PGC)-like cells. (A) Immunofluorescence staining (merged DAPI and primary antibody-second antibody-FITC stains) of octamer-binding transcription factor 4 (OCT4), VASA, STELLA, interferon-induced transmembrane protein 3 (IFITM3) and deleted in azoospermia-like (DAZL) in PGC-like cells on day 7. (B) Light microscopic images of the same cells. (C) Negative controls obtained by incubating the cells with secondary anti-rabbit or anti-mouse antibody alone after staining with DAPI. Scale bar is set to 50 *μ*m.

OCT4, VASA, STELLA, interferon-induced transmembrane protein 3 (IFITM3) and DAZL were detected in the PGC-like cells on day 7 of induction of differentiation, while no signal was detected in the negative controls with only the anti-rabbit or anti-mouse secondary antibody (Fig. 2).

**Figure 3:**
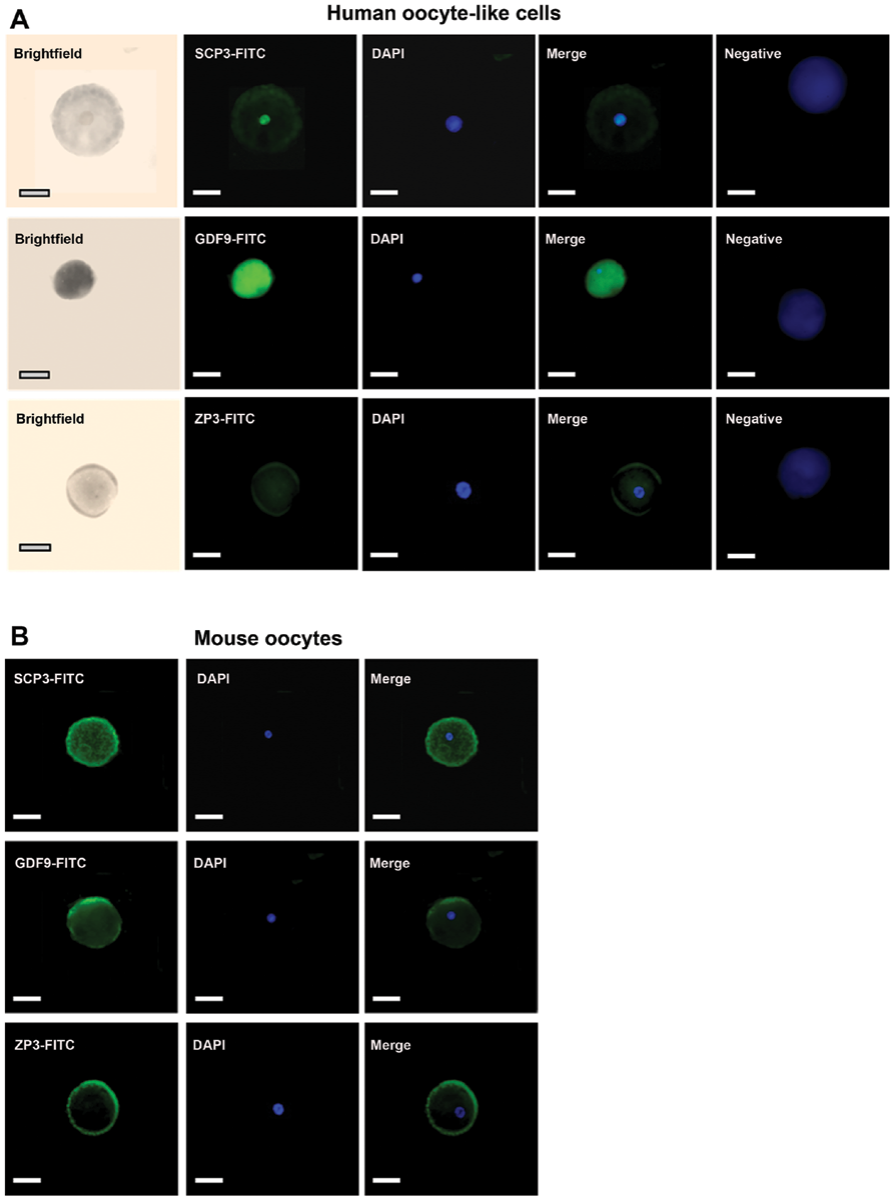
Immunofluorescence staining of synaptonemal complex protein 3 (SCP3), growth/differentiation factor 9 (GDF9) and zona pellucida glycoprotein (ZP)3 in human oocyte-like cells (OLCs) differentiated from human first-trimester umbilical cord (hFTUC)-derived stem cells. (A) Immunofluorescence staining of SCP3, GDF9 and ZP3 in the OLCs. Negative controls were obtained by incubating OLCs with secondary anti-rabbit antibodies alone after staining with DAPI. Note that the DAPI staining of a single germinal vesicle in the human OLCs. The zona pellucida can be detected surrounding the differentiated human OLCs. (B) Immunofluorescence staining of SCP3, GDF9 and ZP3 in mouse oocytes, which were used as positive controls. Scale bar is set to 100 *μ*m.

Furthermore, the OLCs at 24 days of induction were positively stained with anti-SCP3, anti-GDF9 and anti-ZP3 primary antibodies (Fig. 3A). The specificity of the antibodies was identified using mouse oocytes as positive controls as the primary antibodies used in this study all reacted with humans and mice (Fig. 3B). More interestingly, DAPI staining revealed that the OLCs contained a germinal vesicle. This demonstrated that the OLCs were single cells rather than cell aggregations, which also were the same as mouse oocytes (Fig. 3). Notably, ZP-like structures surrounding OLCs could be detected in some of the cells by using anti-ZP antibodies (Fig. 3A), which were very similar to matured mouse oocytes (Fig. 3B).

### RT-qPCR and western blot analysis of specific markers of PGCs and oocytes

To further characterize the PGC-like cells and OLCs, RT-qPCR was performed. In RT-qPCR, the specificity of each amplified product in the controls and differentiated cells were verified by a bi-directional sequence analysis. Once the specificity of the products was established, temporal changes in the relative mRNA levels of each of the markers were evaluated using β-actin as an inner control. The Ct values for all the products were <35, and the efficiencies of the targets and the reference (β-actin) were approximately equal.

**Figure 4:**
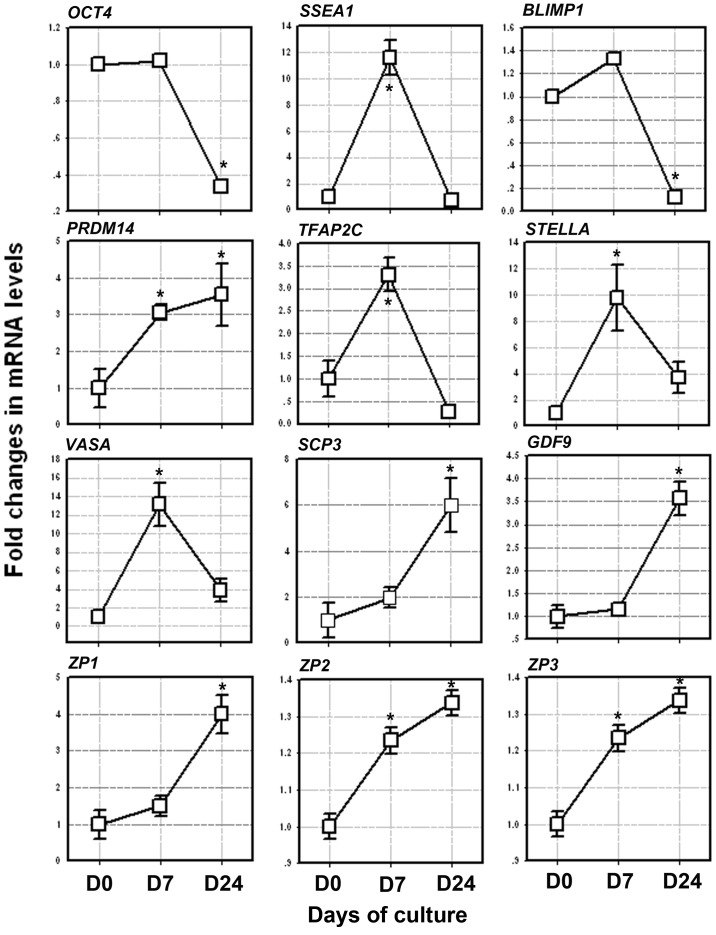
Expression of the germ cell markers, octamer-binding transcription factor 4 (OCT4), stage-specific embryonic antigen 1 (SSEA1), STELLA, VASA, B lymphocyte induced maturation protein 1 (BLIMP1), PR domain containing 14 (PRDM14) and transcription factor AP–2 gamma (TFAP2C), in the primordial germ cell (PGC)-like cells after 7 days of differentiation and the oocyte markers, synaptonemal complex protein 3 (SCP3), growth/differentiation factor 9 (GDF9) and zona pellucida glycoprotein (ZP)1, ZP2 and ZP3, after 24 days of differentiation as determined by RT-qPCR. Relative mRNA levels are normalized for the β-actin housekeeping gene. The results are presented relative to the control cells (2^−ΔΔCt^). Data represent the means ± SEM of 3 independent experiments. *Statistical significance when compared to day 0 of culture.

Transcripts of all the markers were detected in the PGC-like cells and OLCs, as well as in the undifferentiated cell population (Fig. 4). However, fold changes in the differentiated compared to the undifferentiated cells for all the markers showed certain dynamic changes during the induction process. On day 7 of the induction of differentation, in which the PGC-like cells appeared, the mRNA levels of OCT4, SSEA1, STELLA, VASA, BLIMP1, PRDM14 and TFAP2C increased by approximately 1.02-fold (P=0.246), 11.6-fold (P=0.001), 9.8-fold (P=0.001), 13.3-fold (P=0.001), 1.4-fold (P=0.005), 3.3-fold (P=0.01) and 3.05-fold (P=0.0025), respectively, when compared to the levels obtained on day 0 (Fig. 4). As these genes are determinants in the transition of PGCs, their increased expression may indicate initial PCG-like cells during the induction process.

From day 7–24, the mRNA levels of SCP3, GDF9, ZP1, ZP2 and ZP3 gradually increased when compared to the levels obtained on day 0. On day 24 of differentiation, in which the OLCs were developed, the mRNA levels of SCP3, GDF9, ZP1, ZP2 and ZP3 increased by approxiamtely 6-fold (P=0.039), 3.5-fold (P=0.007), 4-fold (P=0.014), 1.33-fold (P=0.036), and 8-fold (P=0.008), respectively, when compared to the levels obtained on day 0 (Fig. 4).

**Figure 5:**
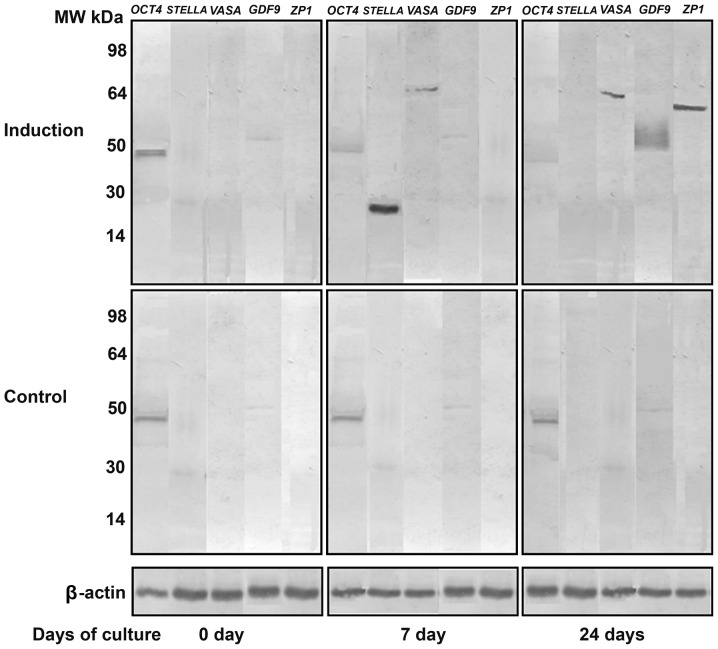
Western blot analysis of the protein expression of octamer-binding transcription factor 4 (OCT4; MW, 45 kDa), STELLA (MW, 23 kDa), VASA (MW, 80 kDa), growth/differentiation factor 9 (GDF9; MW, 51 kDa) and zona pellucida glycoprotein 1 (ZP1; MW, 70 kDa) on day 0, 7 and 24 after the induction of differentiation. Protein expression of STELLA, VASA, GDF9 and ZP1 was not detected in the untreated stems cells except for OCT4. β-actin (MW, 42 kDa) was used as an internal control.

In order to further confirm the results of RT-qPCR, western blot analysis was performed. The protein expression levels of STELLA and VASA on day 7 and those of GDF9 and ZP1 on day 24 after the induction of differentiation were markedly increased, while OCT4 protein expression showed a slight decrease from day 0–24 (Fig. 5). These changes in the protein expression levels were similar to those of the mRNA expression levels.

### Production of estradiol, VEGF and LIF by COC-like cells

**Figure 6:**
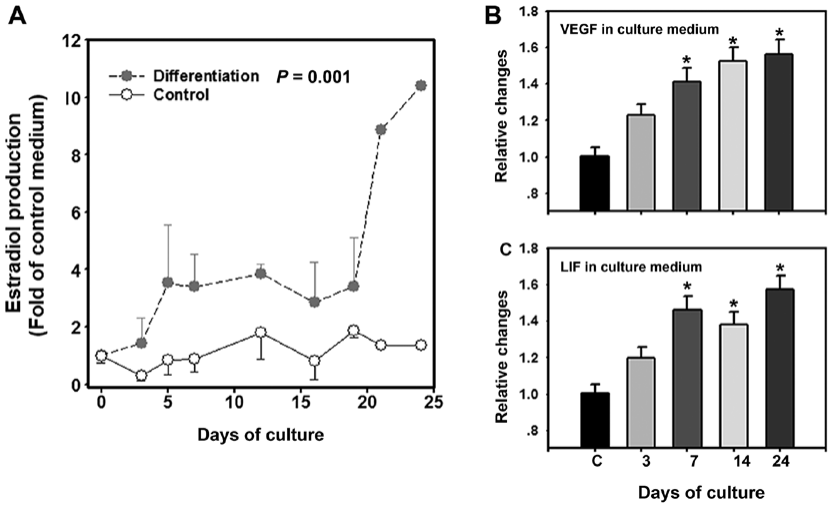
Determination of fold-changes in levels of (A) estradiol, (B) vascular endothelial growth factor (VEGF) and (C) leukemia inhibitory factor (LIF) in the culture medium during the differentiation process. There was a significant difference in the estradiol levels between the differentiated and undifferentiated cells (P<0.001). Concentrations of VEGF and LIF gradually increased from day 0–24 during the differentiation process. All data are represented as the means ± SEM from 3 independent experiment sets. (B and C) ^*^Statistical significance when compared to day 0 of culture. C, control.

Before day 18, there were low levels (ranging from 0.24–2.79 ng/ml) of estradiol in the culture medium of the undifferentiated cells (controls), while the estradiol levels in the culture medium of the cells induced to differentiate were 1-4-fold (ranging from 1.42–8.72 ng/ml) higher than the controls. The estradiol concentrations increased consistently after 18 days, with the levels peaking (22.3–27.9 ng/ml; 8-10-fold increase) on day 21–24 (Fig. 6A). In addition, the concentrations of LIF and VEGF in the culture medium also gradually increased from day 0–24 (Fig. 6B and C). The concentration of LIF in the human follicular fluid (control, C) was 0.91±0.04 ng/l, while the concentrations of LIF in the culture medium were 1.07±0.05 ng/l (day 3), 1.33±0.04 ng/l (day 7), 1.25±0.03 ng/l (day 14) and 1.43±0.03 ng/l (day 24). The concentration of VEGF in the human follicular fluid (control, C) was 410.6±20.5 pg/ml, while the concentrations of VEGF in the culture medium were 503.5±25.1 pg/ml (day 3), 572.4±26.8 pg/ml (day 7), 625.1±30.2 pg/ml (day 14) and 641.2±23.8 pg/ml (day 24) (Fig. 6B and C).

## Discussion

A number of previous studies have demonstrated that PGC-like cells and OLCs can be generated from embryonic, differentiated pluripotent and adult stem cells *in vitro* (2–7,18). In these studies, the induction of embryonic or somatic stem cells into OLCs was generally performed by culturing the cells with growth factors (3,6), estrogenic stimuli (12), conditional medium from testicular cell cultures (19), follicular fluid and gonadotrophins (8), or with ovarian granulose cells (3). In the present study, we demonstrated that stem cells derived from hFTUC also differentiate into PGC-like cells and OLCs by the addition of human follicular fluid, gonadotrophins and estradiol to the culture medium.

We demonstrated that our germ cell precursors closely resembled PGCs or oocytes based on the following factors: i) morphologic changes; ii) marker expression profiles at the mRNA and/or protein level; and iii) the production of estradiol from COC-like structures.

As has been previously demonstrated, germ cell development requires a series of multiple well-orchestrated steps, which involve the concurrent up- and downregulation of the expression of specific genes (20). In the present study, on day 7 of differentiation, the PGC-like cells expressed the proteins OCT4, IFITM3, VASA, STELLA and DAZL (Figs. 2 and 5), which are markers indicative of germ cell formation. In particular, OCT4 has been suggested to be required for PGC survival (20). IFITM3 is believed to initiate the repression of homeobox genes in early germ cell precursors (20), while STELLA plays a role in facilitating germline and endodermal differentiation of human ES cells (21). A lack of STELLA expression at the earlier stage can reflect a transition of cells committing to the germ lineage (19). DAZL is considered essential for PGC development, as knockout mice lack a germ cell population (22,23). VASA is expressed in post-migratory PGCs until the post-meiotic stage of oocytes (24,25).

Moreover, in this study, the mRNA levels of BLIMP1, PRDM14, TFAP2C, SSEA1, STELLA and VASA first increased and then decreased at later stages of the induction of differentiation (Fig. 4). BLIMP1, PRDM14 and TFAP2C are key germ cell determinants for regulating PGC specification (26,27). It has been reported that BLIMP1 binds directly to suppress the epxression of somatic and cell proliferation-related genes, and directly induces TFAP2C expression, which together with PRDM14, initiates the PGC-specific fate (28). Furthermore, it has been reported that human germ cells express SSEA1 (29). These results together with morphologic similarities indicate that some subpopulations of stem cells are able to differentiate into PGC-like cells.

In this study, indeed, after day 7 of differentiation, the PGC-like cells continued to form structures that morphologically resembled primordial follicles, and COC-like cells/OLCs finally appeared in the culture during day 4–24 of the induction of differentiation. The COC-like cells/OLCs express proteins, such as SCP3, GDF9, ZP1 and ZP3 (Figs. 3 and 5) and the mRNA levels of oocyte-specific markers, such as SCP3, GDF9, ZP1, ZP2 and ZP3 increased during the differentiation process (Fig. 4). SCP3 is a meiosis-specific protein (30), while GDF9 is required for normal folliculogenesis (31), and ZP glycoproteins are expressed only in oocytes (32). Taken together, these results indicate that the some of the PGC-like cells are able to further develop into OLCs.

Furthermore, the detection of estradiol production provides evidence of the functional activity of somatic cells in the COCs. Although we added a small amount of estradiol into the culture medium during the induction process, the 8-10-fold increase in the estradiol concentration over the controls on days 21 and 23 was probably due to the COC-like cells rather than the accumulation of exogenous estradiol as the culture medium was changed every 3 days. This is supported by the fact that the protein concentrations of LIF and VEGF in the culture medium increased consistently during the differentiation process even though the follicular fluid contained a certain amount of the two factors. It is known that granulosa cells express a number of growth factors and cytokines, including LIF (33) and VEGF (34), which have been reported to have beneficial effects on mouse (35) or porcine (36) oocyte maturation. Our findings in this study further suggest that differentiated COC-like cells may include functional granulose cells.

It is interesting to determine the reason that hFTUC-derived stem cells are capable of differentiating into germ cell-like cells, COC-like cells and OLCs. In our previous study, the stem cells highly expressed BMP1 and BMP4 (15). As mentioned earlier, BLIMP1 together with PRDM14 and TFAP2C are key germ cell determinants for regulating PGC specification (26,27). Thus, this suggests that hFTUC-derived stem cells may be an ideal cell resource for an *in vitro* model for the investigation of the occurrence of germ cell formation and differentiation.

Moreover, our previous study indicated that stem cells derived from hFTUC exhibited a significantly greater proliferative potential and were more efficient in their *in vitro* differentiation when compared to stem cells derived from term umbilical cords (15). This may be due to the fact that younger sources of adult stem cell populations have a greater proliferative potential and greater plasticity than their older counterparts (37,38). Therefore, it can be assumed that hFTUC-derived stem cells may be more efficient when differentiating into OLCs. ES cells are the primary stem cells that are capable of developing into any type of cells. It has been reported that ES-derived OLCs are produced after an induction of 26 days in a monolayer culture without feeder cells and without the addition of growth factors (2,10). At the same time, skin stem cell-derived OLCs were generated by the addition of 5% porcine follicular fluid, growth factor EGF and hormones, such as ITS, FSH and LH, and by extending the induction from 30 to 50 days (2,10). Compared to ES-derived and skin stem cell-derived OLCs, the hFTUC-derived OLCs in the present study were generated within a shorter time frame (14–24 days); these results are similar to those of a recent study in which human amniotic fluid stem cells generated OLCs within 3 weeks (39).

Overall, our findings support the idea that the hFTUC-derived stem cells have an intrinsic ability to differentiate into PGC-like cells and OLCs. However, whether the OLCs contain synapsed homologous chromosomes or whether the OLCs become embryos after subsequent *in vitro* fertilization are questions that require further investigation in future studies.

Currently, factors regarding on how to induce the up- or downregulation of markers of PGCs and oocytes remain unknown. Human follicular fluid contains a variety of biochemical substances both transferred from the blood plasma and secreted from granulosa and theca cells (40). As oocytes secrete soluble paracrine growth factors that can regulate granulose cell development, and granulosa cells in turn regulate oocyte growth during follicle formation (41), it is apparent that the biochemical substances from the granulosa cells may play a key role in the initiation of germ cell formation and oocyte development. Nonetheless, in order to answer the question of whether these factors, alone or in combination, are responsible for initiating the differentiation of stem cells into germ cell lineages, further studies are required in the future.

In conclusion, in this study, we demonstrated that stem cells derived from hFTUC have an intrinsic ability to differentiate into OLCs. This provides a novel *in vitro* model for the investigation of the mechanisms through which germ cell formation and differentiation occurs. Nevertheless, further studies are warranted in order to fully elucidate the accurate functionality of these stem cells, and which factors are responsible for the initiation of these stem cells into germ cell lineages.

## Figures and Tables

**Table I:** Source and dilution of primary antibodies used in this study.

Antibodies	Clone	Catalog no.	Manufacturer	Dilution
OCT4	Rabbit polyclonal	Ab18976	Abcam, Cambridge, UK	1:200
VASA	Rabbit polyclonal	SC-67185	Santa Cruz Biotechnology, Inc., Dallas, TX, USA	1:100
STELLA	Mouse antibody	Ab74531	Abcam, Cambridge, UK	1:200
IFITM3	Rabbit polyclonal	SC-66827	Santa Cruz Biotechnology, Inc., Dallas, TX, USA	1:100
DAZL	Rabbit polyclonal	SC-36604	Santa Cruz Biotechnology, Inc., Dallas, TX, USA	1:200
SCP3	Rabbit polyclonal	SC-33195	Santa Cruz Biotechnology, Inc., Dallas, TX, USA	1:200
GDF9	Rabbit polyclonal	Ab38544	Abcam, Cambridge, UK	1:100
ZP1	Rabbit polyclonal	Ab171954	Abcam, Cambridge, UK	1:200
ZP3	Rabbit polyclonal	Ab48895	Abcam, Cambridge, UK	1:200

OCT4, octamer-binding transcription factor 4; IFITM3, interferon-induced transmembrane protein 3; DAZL, deleted in azoospermia-like; SCP3, synaptonemal complex protein 3; GDF9, growth/differentiation factor 9; ZP, zona pellucida glycoprotein.

**Table II:** List of primers used in qPCR.

Genes	Primers	Amplified size (bp)
OCT4	CCCACACTGCAGCAGATCAGTTGTGCATAGTCGCTGCTTGA	110
BLIMP1	TGGAGAACGGCCTTTCAAATCCTGGCATTCATGTGGCTTT	110
PRDM14	GAGTCAGGTTTGGGCCCTTTGTGGCTCAAATGACCATCTTCA	110
TFAP2C	TATGTCTGTGAAGCCGAATTTCCGCCGCCAATAGCATGTTCTT	110
SSEA1	CACCAACTGAGCCAACATGTGGCCAGAGCTTCTCGGTGATATAA	160
STELLA	GCGGAGTTCGTACGCATGACCATCCATTAGACACGCAGAAA	110
VASA	TTTCCAAGAGAGGCGGCTATCAGTGCGCTGCATACATTCGT	155
SCP3	TGCAGTCATTGAGAAACGTAGGAGCAAGAAGAGCCTTGTTAATGTCA	110
GDF9	TCTCCAGTTCACACCATGGTACAATCGGGCTCAATGGTCAAAA	110
ZP1	CCGCTTCAAGGTGGTGGATCCTCTGTAATCGGCCGAGAA	110
ZP2	CAGAGGTGTCGGCTCATCTGAGCAGTCTTGTGCCCTTTGGT	110
ZP3	GACCCGGGCCAGATACACTCATCTGGGTCCTGCTCAGCTA	110
β-actin	TGGCATTGCCGACAGGATGGACAGCGAGGCCAGGAT	110

OCT4, octamer-binding transcription factor 4; BLIMP1, B lymphocyte induced maturation protein 1; PRDM14, PR domain containing 14; TFAP2C, transcription factor AP-2 gamma; SSEA1, stage-specific embryonic antigen 1; SCP3, synaptonemal complex protein 3; GDF9, growth/differentiation factor 9; ZP, zona pellucida glycoprotein.
